# Deconstructing eye contact perception: Measuring perceptual precision and self-referential tendency using an online psychophysical eye contact detection task

**DOI:** 10.1371/journal.pone.0230258

**Published:** 2020-03-13

**Authors:** Carly A. Lasagna, Merranda M. McLaughlin, Wisteria Y. Deng, Erica L. Whiting, Ivy F. Tso

**Affiliations:** 1 Department of Psychiatry, University of Michigan, Ann Arbor, Michigan, United States of America; 2 Department of Psychology, University of Miami, Coral Gables, Florida, United States of America; 3 Athinoula A. Martinos Center for Biomedical Imaging, Charlestown, Massachusetts, United States of America; 4 Department of Psychiatry, Massachusetts General Hospital, Boston, Massachusetts, United States of America; Birkbeck University of London, UNITED KINGDOM

## Abstract

Eye contact perception—the ability to accurately and efficiently discriminate others’ gaze directions—is critical to understanding others and functioning in a complex social world. Previous research shows that it is affected in multiple neuropsychiatric disorders accompanied by social dysfunction, and understanding the cognitive processes giving rise to eye contact perception would help advance mechanistic investigations of psychopathology. This study aims to validate an online, psychophysical eye contact detection task through which two constituent cognitive components of eye contact perception (perceptual precision and self-referential tendency) can be derived. Data collected from a large online sample showed excellent test-retest reliability for self-referential tendency and moderate reliability for perceptual precision. Convergence validity was supported by correlations with social cognitive measures tapping into different aspects of understanding others. Hierarchical regression analyses revealed that perceptual precision and self-referential tendency explained unique variance in social cognition, suggesting that they measure unique aspects of related constructs. Overall, this study provided support for the reliability and validity of the eye contact perception metrics derived using the online Eye Contact Detection Task. The value of the task for future psychopathology research was discussed.

## Introduction

Eye gaze is a ubiquitous social cue conveying the attention and intentions of the gazer [1,2]. Thus, gaze perception—the ability to accurately and efficiently discriminate others’ gaze directions (especially self-directed ones)—is critical to deciphering social cues and navigating the complex social world. Gaze perception develops early in life and supports higher-level social functioning [3,4]. Its disruption can lead to social dysfunction, evidenced by reports of abnormal eye contact perception in conditions often accompanied by social deficits, including autism-spectrum disorder (ASD) [5], social anxiety [6–8], schizophrenia [9–11], and bipolar disorder [12]. A relation between gaze processing and the strength of autism, social anxiety, schizotypal traits has also been found in subclinical populations [13–15]. These findings suggest that gaze perception is a functional dimension cutting across categories of mental disorders. Investigating the processes underlying altered gaze perception would advance our understanding of psychopathology and inform treatment, consistent with the goal of the Research Domain Criteria (RDoC) initiative of the National Institute of Mental Health within the United States [16]. To advance this research, we need a test that can deconstruct the cognitive processes giving rise to eye contact perception and at the same time easily administered to increase the accessibility among researchers. Towards this end, we have developed an online tool to measure perception of *self-directed* gaze (referred to as ‘eye contact perception’ hereafter) using psychophysics to disentangle underlying mechanisms; the current paper aims to assess the reliability and validation of this tool.

Eye contact perception relies on basic sensory processing of visual stimuli (i.e., perceiving eyes in the context of a face) and a higher-level cognitive component (i.e., judging whether gaze is self-referential or not). The former (called ‘perceptual precision’ hereafter) is a low-level process, whereby the visual system encodes sensory information such as the position of the iris relative to the sclera or the orientation of the head. The latter (called ‘self-referential tendency’ hereafter) is a higher-level cognitive process through which prior knowledge and beliefs are integrated with sensory data to form perception and, ultimately, facilitate the categorization of eye gaze as self-directed or not. “Normal” eye contact perception thus requires both intact visual processing and appropriate self-referential processing. Disruption to either of these processes could result in atypical gaze perception. For example, temporary or situation-dependent factors that add perceptual noise (e.g., due to distance or lighting intensity [17]) can reduce the precision of gaze perception, and those that evoke a self-related belief (e.g., when one’s name is being called [18]) can increase the likelihood of perceiving self-directed gaze. Additionally, in psychiatric disorders in which abnormal gaze perception is well-documented (e.g., schizophrenia, ASD, and social anxiety), a large body of evidence also suggests disruptions in either/both visual perception and/or self-referential processing (e.g., dysfunction in low-level visual processing in ASD [19]; dysregulated self-referential tendencies in social anxiety [20,21]; deficits in both low-level visual processing [22,23] and higher-level self-referential processes [24] in schizophrenia). Therefore, dissociating these two cognitive processes can help us identify the sources of deficits underlying abnormal gaze perception in different psychiatric disorders as well as understand individual differences across the health-psychopathology continuum.

To better understand these two crucial processes, it is necessary to employ methods designed to disentangle precision (or sensitivity) and overall tendency (or bias) during gaze processing. Psychophysics methods offer a promising solution because they are used extensively in basic sensory processing research to achieve this exact goal. In a traditional psychophysics experiment, stimulus signal strength is systematically manipulated so that a psychometric function relating the observer’s response to signal strength can be fitted to data. This enables the estimation of two important perceptual properties: threshold and slope [25]. Using a yes-no detection task, threshold of the psychometric curve references the signal strength that elicits positive responses 50% of the time, indexing the signal strength needed by the observer to meaningfully detect the signal. The slope (when detection rate = 50%) of the curve indexes the sensitivity of the sensory system to discriminate ambiguous stimuli. Eye contact perception mirrors signal detection of sensory stimuli, making it an ideal process to be studied using psychophysics. In fact, psychophysical studies of eye contact perception could be dated back to as early as 1963 [26], with measurement and statistical methods refined in more recent studies [11,12,27]. In a psychophysical eye contact detection task (e.g., [11,12,26–28]), observers view actors depicting a range of gaze angles in gradual increments from “looking at me” to “looking away from me,” and must indicate whether or not the person is looking at them. Researchers manipulate signal strength through the gaze angles portrayed by actors: when the eye contact signal is strongest, the actor appears to look directly at the viewer; when the signal is weakest, the actor’s eyes are turned completely away from the viewer; and most importantly, at ‘intermediary gaze angles’, when the signal strength is only moderate, it is not always clear to the viewer whether the actor is looking at them or not. In studies of this kind, a psychometric function can be fitted to the eye contact endorsement data. The threshold and slope of the psychometric function can then be used to quantify self-referential tendency and perceptual precision [11,12], whereby threshold reflects the subjective bias in perceiving eye contact (i.e., self-referential tendency) and slope indicates how rapidly perception changes from absence to presence of eye contact detection with respect to signal strengths (i.e., perceptual precision).

Despite their merits, psychophysics studies are not widely utilized among published articles on gaze processing. Some previous studies include no intermediary gaze angles [29,30] and, instead, use only faces *clearly looking at* and *clearly looking away from* the viewer and quantify eye contact perception as accuracy of categorization. This approach is unable to offer insight into the cognitive mechanisms and often insensitive to individual/group differences that emerge only when the eye contact signal is moderate in strength (for detailed discussion, see [11]). In view of this, some studies used more gaze angles and tested for group differences in proportion of eye contact endorsements at each of the gaze angles and their associations to external measures [6,26]. Better yet, a number of more recent studies [13,15,31–33] used broad, continuous ranges of gaze angles to better characterize eye contact perception, for example, to measure the range of gaze directions that observers feel looked at (i.e., the Cone of Direct Gaze; CoDG [31]), and examine its clinical or behavioral correlates. While the CoDG has been shown to be associated with a number of psychopathology traits [6,13,33], there have been inconsistencies in its interpretation. Some have interpreted a wider CoDG as indicative of stronger self-referential tendency [13,31,34], while others have attributed it to poorer precision [35]. Notably, Mareschal et al. (2013) showed that while the CoDG width is unaffected by visual noise, the variance in the width is increased. This suggests that the CoDG width is likely an index of self-referential bias, while the *variance* in the CoDG width (which is analogous to the slope of the psychometric function of eye contact perception) an index of visual perceptual precision.

Another issue with previous eye contact perception studies deals with sample size. Most previous studies have used small samples, with the majority consisting of fewer than 20 participants (e.g., [9,18,30,36–39]) and a significant portion using samples of 10 or less (e.g., [17,26,31,33,35,40]). Besides limiting the generalizability of the findings, small sample sizes also preclude the investigation of important questions about eye contact perception, such as within-subject reliability over time, relationships to other social cognitive functions, sex differences, and age effect. The problem of small sample sizes may be addressed by increasing the ease of administration and access of eye contact perception paradigms through online experimentation. Well-designed online studies enable the collection of data easily and cost-effectively across time points, contexts, and large, diverse samples. Take, for instance, the well-known Implicit Association Test (IAT; projectimplicit.org), which transitioned to a web-based interface in 1998 and within two years, data was collected from over 600,000 respondents, expediting scientific knowledge of implicit biases [41]. Online adaptation of cognitive experiments would also make tasks available outside of specialty software (e.g., MATLAB, E-Prime), which is often not accessible to smaller research labs with limited resources. Therefore, if a rigorous psychophysical eye contact perception task can be easily and reliably administered online, it would accelerate the progress in this research area. Recently, Schulze et al. [6] conducted an eye contact detection study consisting of nearly 300 trials online. They successfully collected data from more than 200 participants and found that the proportion of direct gaze endorsements was positively associated with higher level of social anxiety. Although the authors did not analyze the data (collected using 5 different gaze angles) using a psychophysical approach, their study provides preliminary support for the feasibility of online administration of *psychophysical* eye contact perception research that typically requires participants to complete a large number of trials.

### The present study

The current study presents an online adaptation of a psychophysical Eye Contact Detection Task, adapted from Tso and colleagues [11], with the goal of validating this online tool for future investigations of the mechanisms of altered eye contact perception in psychiatric disorders and across individuals. The Eye Contact Detection Task presents face images to participants, who must indicate whether they feel that the face is looking at them or not. Stimuli cover 11 eye contact signal strengths from eyes averted 30° away from the viewer (signal strength = 0) to direct eye contact (signal strength = 1), in gradual increments and presented in two head orientation conditions (forward: head facing the viewer; deviated: head turned 30° away from the viewer). Head orientation was manipulated because it is a key factor known to influence the perception of eye contact [39,42,43]. The task is completed through an online survey platform, accessible using any internet browser and requiring no specialty software. Data was collected from a large online sample to assess feasibility, validity, and reliability of online administration. Participants completed the Eye Contact Detection Task, as well as a battery of previously-validated tests of social cognition and self-report psychological measures. Four weeks later, a subset of the participants completed the Eye Contact Detection test again, to assess test-retest reliability. We hypothesized that the eye contact perception metrics (perceptual precision and self-referential tendency) would show good test-retest reliability and convergent validity. Given evidence suggesting that men are more likely than women to perceive gaze as self-directed [15,44] and that women often score higher than men on tests of social cognition [45,46], we examined sex differences in the eye contact perception metrics in the current study. Finally, changes in eye contact perception over the lifespan are largely unaddressed in the existing literature, so age was also explored as a measure of interest.

## Methods

### Participants

The present study recruited an online sample of (*N =* 300) English-speaking adults from the United Kingdom, United States, and Canada to participate in Phase I. To ensure a balanced distribution of sex and age (participants recruited online are known to skew towards younger demographics), we limited recruitment to 50 men and 50 women from each of three age groups: 18–25, 26–45, and 46–60. Four weeks later, a subset of participants from the initial sample (20 men and 20 women from each age group; *N* = 120) were invited back to complete Phase II of the study to assess test-retest reliability. Complete demographic information for the full Phase I sample is presented in Table 1. Demographic characteristics of the sample broken down by age group are provided in S1 Table.

**Table 1:** Sample characteristics at Phase I.

	Male (*N =* 151)	Female (*N* = 148)	All (*N =* 299)
	M (SD)	M (SD)	M (SD)
Age	34.8 (13.2)	35.9 (13.0)	35.4 (13.1)
Education	14.7 (2.3)	14.9 (2.3)	14.8 (2.3)
Parental Education	14.3 (2.7)	13.7 (2.5) ^a^	14.0 (2.6) ^a^
Race/Ethnicity	**% (*n*)**	**% (*n*)**	**% (*n*)**
* White*	85.4 (129)	84.5 (125)	84.9 (254)
* Black*	4.0 (6)	4.7 (7)	4.3 (13)
* Asian*	9.9 (15)	8.8 (13)	9.4 (28)
* Other/NR*	0.7 (1)	2.0 (3)	1.3 (4)

Based on data from the full sample at Phase I (*N =* 299: 151 males, 148 females). Data was collected for one additional male than was originally intended (resulting in 151, rather than 150 male participants) because of a technical error that occurred on the crowdsourcing website. NR = Prefer not to respond; Education = years of education completed; Parental education = highest education completed by either parent (in years).

^a^ Data on parental education missing for one female participant.

At Phase I, one (*n =* 1) participant was excluded from *all analyses* due to inattention and two (*n =* 2) were excluded from *deviated face analyses only* due to extremely low eye contact endorsement during the deviated face condition. This resulted in a final sample *N =* 299 participants in the forward face condition and *N =* 297 participants deviated face at Phase I. At Phase II, three participants (*n =* 3) out of 120 were excluded due to random responses and/or unusually frequent endorsement across eye contact signal strengths, resulting a sample of *N* = 117 participants with valid data in both forward and deviated face conditions for the reliability analysis.

### Procedure

Participants were recruited through Prolific Academia (PA; www.prolific.ac), an online crowdsourcing platform used by academic researchers to recruit human participants and collect data for scientific research. Prolific users were shown description of the study and those interested in participating were given a link to the study which ran on Qualtrics online survey platform (Provo, UT). Before participating, participants were given details about the study, privacy protections, compensation, and study team contact information. Participants that responded “Yes” to “I have read the above information and agree to participate” began participating at that time.

During Phase I, participants completed an Eye Contact Detection Task, a battery of previously-validated measures of social cognition and psychological traits, and demographic questions. Participants were instructed to complete the study in one sitting using a computer (no tablets, phones, or other electronic devices) in a distraction-free environment. Participants were asked to place themselves directly in front of their computer screen and maximize their browser in order to view images comfortably. Throughout participation, progress was displayed on a progress bar at the top of the page. Those who submitted quality work at Phase I were compensated $7.50 for a median of 45 minutes spent. Four weeks later, 20 men and 20 women from each age group were randomly chosen to complete Phase II of the study. In Phase II, participants completed the Eye Contact Detection Task a second time, in order to assess test-retest reliability. Task presentation and instructions were the same as in Phase I. Participants were compensated $3.20 each and the median time spent was 22 minutes. This study received exempt status from the University of Michigan Institutional Review Board given that identifiable information was not collected and the risk involved was minimal.

### Measures

#### Eye contact detection

The Eye Contact Detection Task used in the present study is an online adaptation of the one reported in Tso et al [11]. Through the Qualtrics online survey platform, participants were presented with static, color images of six actors with neutral facial expressions exhibiting a range of left/right gaze eccentricities. For each face, participants were asked “Is this person looking at me?” and selected a “Yes” or “No” button at the bottom of the screen to respond. Once a response was selected, participants pressed a “Next” button to proceed to the subsequent trial. Participants were encouraged to use their first impression to respond—to more realistically portray the rapid nature of decision-making inherent in eye contact perception in daily life—but no time restraint was imposed to avoid lost data due to time-out. Images were presented in pseudorandom order so that no same actor appeared in consecutive trials, in order to prevent the illusion of eye movement. Each image was 322 x 400 pixels, though the appearance and size of this image may have varied across computer screens.

**Fig 1:**
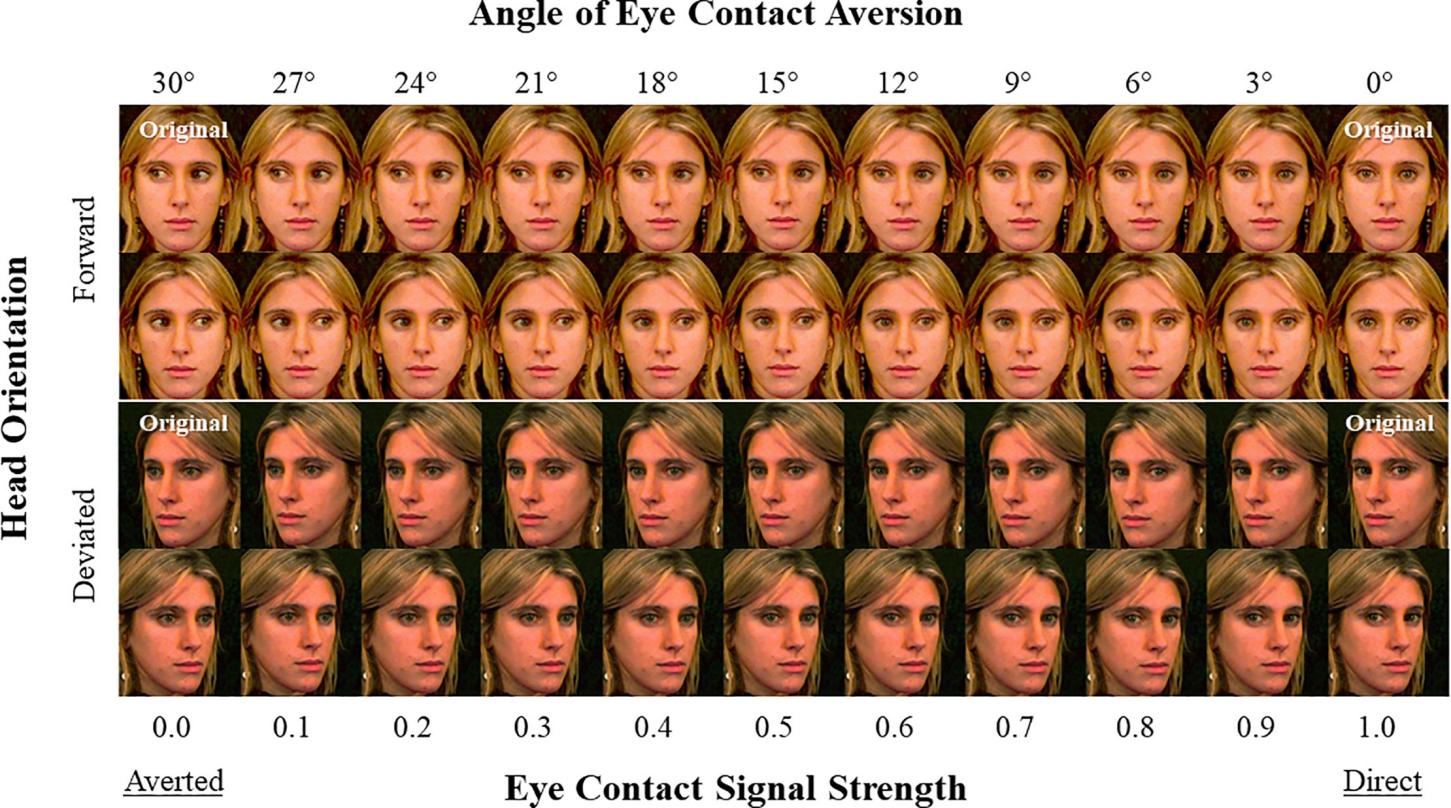
Sample stimuli from Eye Contact Detection Task. Example of stimuli for one actor in the Eye Contact Detection Task. Gaze angles ranged from 30° (averted; signal strength = 0) to 0° (direct; signal strength = 1) in ten 10% increments. The task used face stimuli with both forward (top 2 panels) and deviated (bottom 2 panels) head orientations.

Stimuli were created by morphing a set of original images from George, Driver and Dolan [47] using Abrosoft Fanta Morph software (Beijing, China). Full details of the image-morphing process are provided in S1 File. The final stimuli set consisted of 264 color images, exhibiting 6 actors (3 male, 3 female) × 2 head orientations (forward, deviated) × 11 eye contact signal strengths (eyes averted 0°, 3°, 6°, …, 30°) × 2 gaze directions (eyes averted: leftward, rightward). Examples of original/morphed images for one actor are provided in Fig 1. For analyses, left/right gaze directionality were collapsed within signal strengths, resulting in 12 stimulus presentations per signal strength within each head orientation condition.

#### Social cognition

*Penn emotion recognition task (ER-40)*. The ER-40 [48] measures the ability to perceive and interpret basic, prototypical emotions (happiness, sadness, anger, fear, and neutral) through facial expression. Forty face images were downloaded from the original ER-40 and presented in a pseudorandom order in this study, with no same actor or emotion appearing consecutively. Basic emotion recognition ability was indexed by the overall accuracy score.

*Reading the Mind in the Eyes (RME)*. The RME test [49] measures recognition of complex emotions and mental states. The RME test has been validated in both healthy adults and psychiatric populations with social cognitive deficits [49–52]. During the test, participants view 36 images showing only the eye region of actors. Each image is accompanied by four adjectives (e.g., curious, apologetic, confused, relieved), and participants are asked to choose the word that best describes the actor’s mental state. The original RME is a paper-and-pencil test, and participants are provided with a list of the definition of each word used in the test. We adapted the original test to a computerized format online, such that the definition of each word and an example of it being used in a sentence are shown when the cursor is hovered over the word. Participants were informed of this as part of test instructions. This was arranged to reduce the effort to look up the meaning of each adjective used in the test, thereby reducing the effect of vocabulary on performance, which has been documented [52]. Complex emotion recognition ability was quantified by total accuracy on the RME.

#### Psychological traits

*Questionnaire of Cognitive and Affective Empathy (QCAE)*. The QCAE is a 31-item self-report scale used to measure cognitive empathy (the ability to understand others’ emotions) and affective empathy (the ability to imagine the emotional experiences of others; [53]). Participants are presented with statements and were asked to determine how strongly they agree with them on a 4-point Likert scale, with higher scores indicating higher levels of empathy.

*Autism Spectrum Quotient (ASQ)*. The ASQ is a 50-item questionnaire measuring the degree of autism traits present in an adult with normal intelligence [54]. Half of the items describe behaviors that are typical along the autism spectrum, while the other half describe behaviors often lacking in individuals with autism. Participants responded to each question by choosing one of four options: “definitely agree,” “slightly agree,” “slightly disagree,” or “definitely disagree” (no neutral choice available). Endorsements of autistic traits (“definitely agree” or “slightly agree”) or disagreements of behaviors lacking in autism (“definitely disagree” or “slightly disagree”) receive one point. Total score ranges from 0 to 50, with higher scores indicating higher levels of autistic traits.

*Referential Thinking Scale (RTS)*. The RTS is a 34-item true/false questionnaire designed to measure the sort of referential thinking that is commonly seen in schizotypy [55] and is distinct from normative heightened self-awareness or self-consciousness as observed in social anxiety [56,57]. Higher scores indicate higher levels of schizotypic traits.

### Derivation of eye contact perception metrics

Data from the Eye Contact Detection Task was processed and analyzed using a psychophysical approach. Gaze angles of face stimuli (30°, 27°, …, to 0°) were converted to a scale of ‘eye contact signal strength’ (0, 0.1, …, to 1.0, respectively), where a strength of 1.0 referenced direct eye contact and 0 referenced stimuli with the most averted gaze. In each trial, a participant made a binary (yes or no) response to indicate their perception of self-directed gaze. Therefore, the observed number of “yes” responses, *y*, for each eye contact signal strength, follows a binomial probability distribution dependent on *μ* (an unknown value underlying the probability) and *T = 12* (the number of trials). In our previous work, eye contact detection rates across signal strengths have been shown to follow a logistic pattern [11]. Therefore, we used a logit function to link *μ* to eye contact signal strength *X* using two free parameters, *b*_*0*_ and *b*_*1*_:

$$\log\frac{\mu }{1-\mu }=b_{0}+b_{1}X$$ (1)

**Fig 2:**
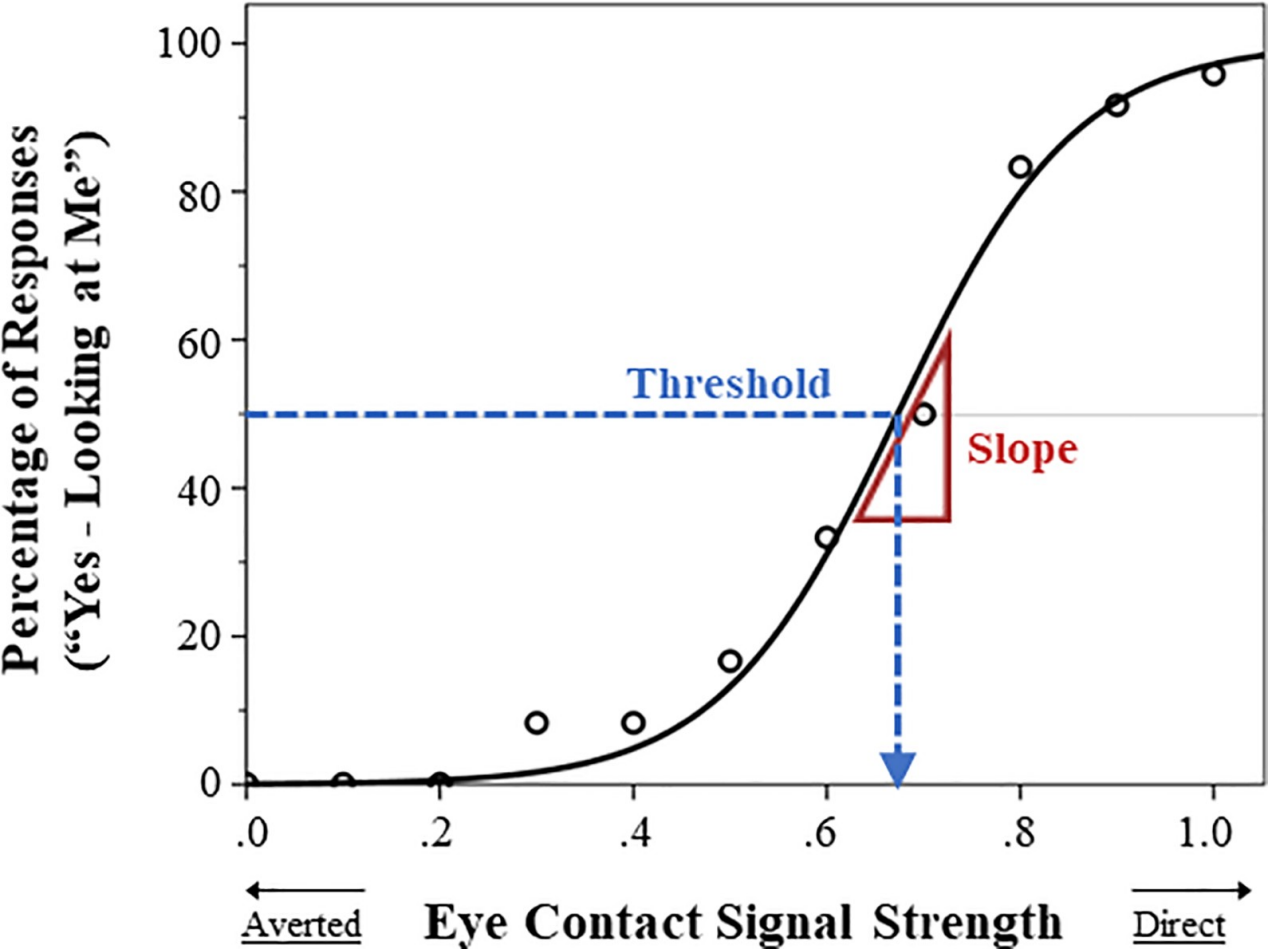
Sample curve-fitting for Eye Contact Detection Task data. A logistic function was fitted to each participant’s Eye Contact Detection Task data (separately for forward and deviated faces) in order to derive two measures of eye contact perception: *self-referential tendency* (threshold at 50% eye contact endorsement) and *perceptual precision* (slope at *y* = 50%).

We used generalized linear model regression and maximum likelihood estimation, as implemented in MATLAB (R2019a) using the function ‘glmfit’, to estimate *b*_*0*_ and *b*_*1*_ for each participant. These two parameters were used to compute threshold (*-b*_*0*_ */ b*_*1*_) and slope (*b*_*1*_*/4*), to respectively index *self-referential tendency* and *perceptual precision*. This was done separately for each head orientation (see Fig 2). This resulted in 4 measures of eye contact perception for each participant: perceptual precision for forward (slope-forward) and deviated faces (slope-deviated), and self-referential tendency for forward (threshold-forward) and deviated faces (threshold-deviated).

Goodness of fit was assessed through examination of deviance residuals and visual inspection (procedure outlined by Vida and Maurer, [58]; [25]). Residual deviance approximately follows a χ^2^ distribution and a χ^2^ probability < .05 is often interpreted as an indicator of poor fit [58]. This method identified a number of participants with poor fitting (i.e., high deviance) for forward (*n =* 7) or deviated faces (*n =* 22). Visual inspection revealed a number of participants (forward: *n =* 10; deviated: *n* = 13) with overfitted data (i.e., deviance approaching zero), all of whom had extreme slope estimates. After removing these participants, the final samples for subsequent analyses were: *N =* 282 for forward faces and *N =* 269 for deviated faces at Phase I; and *N =* 110 for forward faces and *N =* 97 for deviated faces for test-retest reliability analyses (descriptive statistics of deviance residuals for the participants included in subsequent analyses are provided in S2 Table).

### Statistical analyses

Statistical analyses were conducted using IBM SPSS Statistics (Version 24) with *p <* .05 as an alpha level for significance unless indicated otherwise.

#### Effects of head orientation, sex, and age

To examine the influence of head orientation and participants’ sex on eye contact perception, separate mixed model ANOVA’s with head orientation (forward, deviated) as a within-subjects factor and sex (male, female) as a between-subjects factor were conducted for perceptual precision (slope) and self-referential tendency (threshold). Pearson correlations were also used to explore associations between age and eye contact perception. Due to the exploratory nature of these tests, correction for multiple comparisons using the Benjamini-Hochberg procedure keeping the false discovery rate (FDR) below .05 [59] was implemented to reduce Type I error.

#### Test-retest reliability

Test-retest reliability of eye contact perception measures for forward and deviated faces were assessed over a 4-week duration. For participants who completed Phase I and Phase II (and with valid gaze perception measures at both time points), intra-class correlation coefficients (ICC) based on absolute agreement were calculated for all eye contact perception measures.

#### Convergence validity

To evaluate convergence validity of gaze perception measures, Pearson correlations were conducted to assess their relations with measures of social cognitive ability and related psychological traits. We corrected for multiple comparisons using the Benjamini-Hochberg procedure; since these correlations were hypothesized *a priori*, we used a false discovery rate (FDR) of .10 to strike a balance between Type I and Type II errors.

To examine whether the two types of metrics of eye contact perception (slope, threshold) explained unique variance in higher-level social cognitive ability (ER-40, RME), hierarchical regression analyses were performed. Specifically, eye contact perception measures were added one by one to models in order of correlation strength with the dependent variable. If the full model explained significantly more variance than the reduced model, the predictor was retained; if not, the predictor was removed. This was done until all eye contact perception measures were tested for ER-40 and RME.

## Results

**Fig 3:**
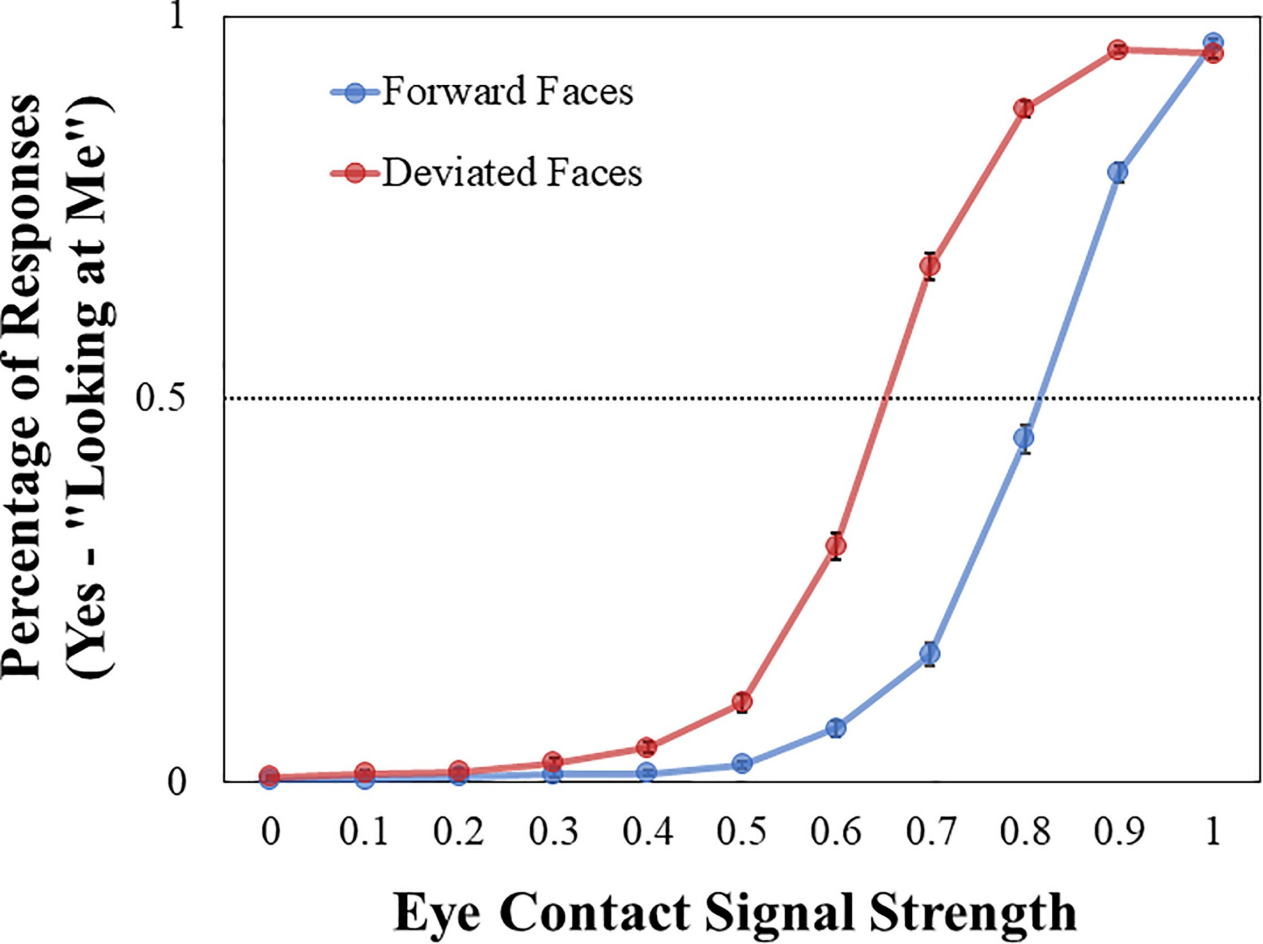
Eye contact endorsement rates across signal strengths. Mean percentage of “Yes–looking at me” responses plotted against eye contact signal strength, calculated separately for forward/deviated faces. Error bars shown represent standard error.

Average eye contact endorsement rates (i.e., percentage of “yes—looking at me” responses) across signal strengths in forward and deviated conditions for the full sample at Phase I are illustrated in Fig 3. Descriptive statistics for the psychophysical eye contact perception measures, as well as the social cognitive/psychological measures, are provided in Table 2 (descriptives further broken down by age group are provided in S1 Table and S3 Table).

**Table 2:** Descriptives for measures of eye contact perception, social cognition, and related psychological traits.

	Male		Female		All	
	*n*	M (SD)	*n*	M (SD)	*n*	M (SD)
*Eye Contact Perception Measures*						
**Forward Faces**						
Perceptual Precision (Slope)	141	5.3(1.7)	141	5.9(1.8)	282	5.6(1.8)
Self-Referential Tendency (Threshold)	141	0.8(0.1)	141	0.8(0.1)	282	0.8(0.1)
**Deviated Faces**						
Perceptual Precision (Slope)	135	4.9(2.3)	134	4.8(2.0)	269	4.8(2.2)
Self-Referential Tendency (Threshold)	135	0.7(0.1)	134	0.7(0.1)	269	0.7(0.1)
*Social Cognition Measures*						
RME	151	27.8 (4.4)	147	28.7 (4.3)	298	28.2 (4.4)
ER-40	151	32.9 (3.3)	147	33.3 (3.0)	298	33.1 (3.1)
*Psychological Measures*						
ASQ	151	20.7 (6.8)	147	20.5 (7.1)	298	20.6 (6.9)
RTS	151	4.8 (5.6)	147	6.6 (5.4)	298	5.7 (5.6)
QCAE	151	89.7 (10.4)	147	94.5 (12.1)	298	92.1 (11.5)

Based on data from the full sample at Phase I (*N =* 299: 151 male, 148 female). ER-40 = Penn Emotion Recognition Task (accuracy); RME = Reading the Mind in the Eyes Test (accuracy); ASQ = Autism Spectrum Quotient (total score); QCAE = Questionnaire of Cognitive and Affective Empathy (total score); RTS = Referential Thinking Scale (total score).

### Effects of head orientation, sex, and age

Results of a mixed model ANOVA revealed a significant effect of head orientation on perceptual precision (slope), *F*(1, 254) = 42.576, *p* < .001, η_p_^2^ = .144, such that it was higher for forward faces (*M* = 5.63, *SD* = 1.77) than deviated ones (*M* = 2.80, *SD* = 2.11). Sex effect on perceptual precision (slope) was not significant, *F*(1, 254) = .452, *p* = .502, η_p_^2^ = .002, but there was a significant interaction between head orientation and sex, *F*(1, 254) = 4.884, *p* = .028, η_p_^2^ = .019. This was driven by higher perceptual precision in men (*M* = 4.87, *SD* = 2.25) than women (*M =* 4.73, *SD =* 1.97) for deviated faces (slope-deviated), but higher perceptual precision in women (*M* = 5.84, *SD* = 1.78) than men (*M* = 5.42, *SD* = 1.73) for forward faces. The main effect of head orientation and the interaction with sex remained significant after correction for multiple comparisons (BH procedure for 3 tests; FDR = .05).

For self-referential tendency (threshold), a similar head orientation effect was also found, *F*(1, 254) = 620.050, *p* < .001, η_p_^2^ = .709, such that self-referential tendency was significantly lower for forward faces (*M* = 0.84, *SD* = 0.10) than for deviated ones (*M* = 0.69, *SD* = 0.11). Neither the main effect of sex, *F*(1, 254) = 1.702, *p* = .193, η_p_^2^ = .007, nor the interaction between head orientation and sex was significant, *F*(1, 254) = .043, *p* = .836, η_p_^2^ = .000, indicating that men and women had comparable self-referential tendency in both forward (female: *M* = 0.85, *SD* = 0.11; male: *M =* 0.83, *SD =* 0.10) and deviated face conditions (female: *M* = 0.69, *SD* = 0.10; male: *M =* 0.68, *SD =* 0.12).

Correlational analyses revealed significant relations between age and eye contact perception measures such that older participants tended to have higher perceptual precision (slope-forward: *r* = .132, *p* = .027; slope-deviated: *r* = .130, *p* = .033) and higher self-referential tendency (threshold-forward: *r* = -.124, *p* = .037) (see S1 Fig for scatterplots depicting these associations). No association was found between age and self-referential tendency for deviated faces (threshold-deviated; *r* = .018, *p* = .764). However, after correcting for multiple comparisons, none of the relationships between age and eye contact perception measures remained significant (BH procedure with FDR critical value = .05; 4 tests).

### Test-retest reliability

Test-retest reliability was assessed using a sample of (*N* = 110) participants for forward faces and (*N* = 97) participants for deviated faces. Complete results are provided in Table 3. Self-referential tendency (threshold) showed excellent ICC for forward (.917) and deviated (.882) faces. ICC of perceptual precision (slope) was fair for forward (.677) and deviated (.629) faces.

**Table 3:** Test-retest reliability (intra-class correlation) of measures obtained from Eye Contact Detection Task.

	Forward Faces (*n* = 110)	Deviated Faces (*n* = 97)
Perceptual Precision (Slope)	.677	.629
Self-Referential Tendency (Threshold)	.917	.882

### Convergence validity

Correlations between eye contact perception measures and other social cognitive and psychological measures are presented in Table 4 (scatterplots in S2 Fig). In general, all 4 eye contact perception measures were significantly associated with both social cognitive measures (ER-40 and RME), such that higher perception precision (higher slope) or lower self-referential tendency (higher threshold) were linked to better recognition of basic and complex emotions. The only exception was self-referential tendency for deviated faces (threshold-deviated), which was associated with ER-40 but not RME.

**Table 4:** Pearson correlations between measures of eye contact perception, social cognition, and related psychological constructs.

	*Eye Contact Perception Measures*			
	Slope		Threshold	
	Forward Faces (*n* = 281)	Deviated Faces (*n* = 269)	Forward Faces (*n* = 281)	Deviated Faces (*n* = 269)
*Social Cognition Measures*				
ER-40	.235***	.237***	.279***	.129*
RME	.311***	.377***	.167**	.026
*Psychological Measures*				
ASQ	-.111	-.036	-.140*	-.060
RTS	-.053	-.139*	-.026	-.032
QCAE	-.056	-.075	-.126*	-.041

Slope = perceptual precision during eye contact detection; threshold = self-referential tendency during eye contact detection (higher thresholds indicate lower self-referential tendency); ER-40 = Penn Emotion Recognition Task (accuracy); RME = Reading the Mind in the Eyes Test (accuracy); ASQ = Autism Spectrum Quotient (total score); QCAE = Questionnaire of Cognitive and Affective Empathy (total score); RTS = Referential Thinking Scale (total score). Asterisks indicate uncorrected *p*-values: *** *p* < .001 **p < .01 *p < .05. All correlations with asterisks remained significant at FDR of < .10. Identical results were obtained with non-parametric Spearman correlations.

Eye contact perception measures were generally more weakly associated with psychological measures than with the two social cognitive measures. The directions of correlations indicated that higher perceptual precision (higher slope) and lower self-referential tendency (higher threshold) were linked to less autistic traits (ASQ), schizotypic referential thinking (RTS) and empathy (QCAE), although only a few reached statistical significance.

As expected, gaze perception measures were moderately correlated with one another (see S4 Table and S2 Fig). To understand whether slope and threshold are able to explain unique variance in social cognition, we used hierarchical regression analyses and the results are shown in Table 5. For ER-40, self-referential tendency for forward faces (threshold-forward) was the strongest correlate and was entered first into the regression model (Model 1), explaining 7.8% of the variance. Perceptual precision for deviated faces (slope-deviated) was the second strongest correlate and was added to the model (Model 2), explaining a significant amount of additional variance (2.0%). Neither of the two remaining eye contact perception measures (slope-forward and threshold-deviated) were able to explain additional variance (Models 3a and 3b, respectively).

**Table 5:** Hierarchal regression on ER-40 and RME with eye contact perception measures as predictors.

Model/Predictor	Model Statistics				Variable Statistics		
	*R*^*2*^	*ΔR*^*2*^	*ΔF*	*p*	*β (s*.*e*.*)*	*t*	*p*
**ER-40 Models**							
*Model 1*							
Threshold-Forward	.078	--	23.633	< .001	8.323 (1.712)	4.861	< .001
*Model 2*							
Threshold-Forward	.090	.020	5.640	.018	6.461 (1.863)	3.469	.001
Slope-Deviated					.218 (.092)	2.375	.018
*Model 3a*							
Threshold-Forward	.102	.011	3.223	.074	5.103 (2.003)	2.548	.011
Slope-Deviated					.153 (.099)	1.546	.123
Slope-Forward					.227 (.126)	1.795	.074
*Model 3b*							
Threshold-Forward	.091	.001	.149	.700	7.014 (2.352)	2.982	.003
Slope-Deviated					.213 (.093)	2.286	.023
Threshold-Deviated					-.794 (2.060)	-.386	.700
**RME Models**							
*Model 1*							
Slope-Deviated	.142	--	44.252	< .001	.767 (.115)	6.652	< .001
*Model 2*							
Slope-Deviated	.149	.021	6.311	.013	.593 (.137)	4.332	< .001
Slope-Forward					.411 (.164)	2.512	.013
*Model 3a*							
Slope-Deviated	.151	.002	.560	.455	.605 (.138)	4.387	< .001
Slope-Forward					.461 (.177)	2.607	.010
Threshold-Forward					-2.096 (2.802)	-7.48	.455
*Model 3b*							
Slope-Deviated	.149	.000	.133	.716	.591 (.137)	4.306	< .001
Slope-Forward					.425 (.168)	2.526	.013
Threshold-Deviated					-.850 (2.332)	-.365	.716

Delta R-squares for Models 2 were relative to Model 1, and those for Models 3 were relative to Model 2. ER-40 = Penn Emotion Recognition Task (accuracy); RME = Reading the Mind in the Eyes Test (accuracy); Slope-forward or slope-deviated = perceptual precision during eye contact detection for forward or deviated faces, respectively; threshold-forward or threshold-deviated = self-referential tendency during eye contact detection for forward or deviated faces, respectively.

For RME, perceptual precision for deviated faces (slope-deviated) was the strongest correlate and was entered first into the regression (Model 1), explaining 14.2% of the variance. Next, perceptual precision for forward faces (slope-forward) was the second strongest correlate and was entered in the next step (Model 2) explaining a significant amount of additional variance (2.1%). Neither of the two remaining eye contact perception measures (threshold-forward and threshold-deviated) were able to explain additional variance (Models 3a and 3b, respectively).

## Discussion

The current paper aimed to establish the online Eye Contact Detection Task as a useful tool for studying the mechanisms of eye contact perception by assessing its feasibility and psychometric properties. Taking advantage of the statistical power afforded by our large dataset, we also explored the potential influences of age and sex on eye contact perception, which had rarely been investigated in the literature.

In terms of feasibility, the Eye Contact Detection Task was successful in its adaptation to an online format, enabling administration online with any internet browser and without special equipment or software. The task, in most cases, took 15–20 minutes to complete. The acceptability of the task was high, as evidenced by high completion rates (i.e., completed by 100% of participants) and high quality of data collected (i.e., < 1% of participants removed due to inattention and/or random responding). Data quality is among the foremost issues faced by researchers using internet-based studies [60,61] and, for internet-based cognitive research, exclusion rates due to response quality are variable, with the low-end at 10–15% of participants (e.g., [62,63]) and the higher-end at 40–50% or above (e.g., [64,65]). The present study removed just 6% to 19% of participants across analyses, well within the lower-end of exclusion rates reported in existing literature. Compared with a previous online eye contact detection study which excluded 23% of participants [6], the exclusion rates of our study were more favorable. Additionally, we replicated the head orientation effect (i.e., stronger self-referential tendency and decreased perceptual precision for deviated faces than forward faces; [11,33]) that has been repeatedly demonstrated in previous laboratory-based studies. Taken together, these findings support that the Eye Contact Detection Task is suitable for use in online (in addition to laboratory-based) studies, and would facilitate large-scale studies in the future, even for labs with minimal equipment or resources.

This study also offered evidence for the stability of eye contact perception measures over time. Test-retest reliability of the threshold, for both forward and deviated faces, over a 4-week interval was in the “excellent” range [66]. This is remarkable given that “measurement noise” associated with environmental factors (e.g., monitor size, screen brightness, position of the participant relative to the screen) could not be controlled because data were collected online. Test-retest reliability of the slope was somewhat lower, in the “fair” range for both forward and deviated faces. One contributing factor may be the uncontrolled within-subjects physical environment across the two time points, as previous psychophysics research has shown that physical factors such as viewing distance, luminance, eccentricity of the retina relative to the stimulus affect the slope of the psychometric curve [38,40]. Interestingly, this finding (that the slope was more sensitivity to sensory noise than was the threshold) is consistent with our theory that the slope reflects bottom-up visual information processing (i.e., perceptual precision), while the threshold was more resilient against sensory noise and likely reflects top-down processing of social information (e.g., prior expectation of self-directed gaze, or self-referential bias).

Another reason for lower test-retest reliability for the slope than the threshold measures may be a mathematical/estimation issue. Specifically, as we can see in Fig 2, participants’ responses were typically all “no” until eye contact signal strength reached a high level, and then responses very rapidly (particularly for forward faces) become mostly “yes”. This response pattern provides few data points around the inflection point of the psychometric curve, which makes the estimation of the parameters sensitive to noise and less stable between occasions. This affects the slope measures more than the threshold measures because, mathematically, the slope is determined by only the *b*_*1*_ parameter; any variability in the *b*_*1*_ estimate due to noise directly translates into variability in the slope measure. In contrast, the value of the threshold is determined by *-b*_*0*_ */ b*_*1*_; these two parameters represent the intercept and slope of a logit function, and their magnitudes tend to go in the same direction (imagine a line with a higher slope *b*_*1*_ would mean a more negative intercept *b*_*0*_), canceling out the effects of noise on the threshold estimates. To improve the reliability of these gaze measures (particularly the slope measures), the range of gaze angles of the face stimuli should be made narrower (e.g., 0° to 15° instead of 0° to 30°) in future studies, so that more data points are available around the inflection point of the curve to improve the accuracy of the parameter estimation. This would also improve the balance between the numbers of “yes” and “no” responses across the task, reducing the confound of motor habituation induced by the predominance of responding “no”. However, this effect should be small in this study because the task design required participants to click the “Next” button after response selection before proceeding to the next trial, which offered a self-correction opportunity if motor habituation occurred.

This study provided preliminary evidence for convergence validity of the eye contact perception measures. Both perceptual precision and self-referential tendency were significantly correlated with performance on recognition of basic and complex emotions. Because the ability to decode others’ facial expressions is key to competence in higher-order socio-emotional functions [48,49,67], our findings indicate that eye contact perception is critical to social functioning not only in clinical populations [11], but also in the general population. It should be noted that, although the ER-40 and the RME have been widely used in social cognition studies (particularly in clinical samples), we are unaware of any previous studies examining their relations to eye contact perception. Thus, the current study also offers a preliminary account of a direct relation between eye contact perception and social cognitive ability as measured using these two well-established tasks.

Relationships between eye contact perception measures and more abstract psychological measures of social processes were also found, though overall weaker and less consistent compared to convergence with perception-based social cognition mentioned above. These associations were generally in the predicted directions. For example, reduced perceptual precision (for deviated faces only) was significantly associated with increased schizotypal referential thinking, which is consistent with a prior report showing an association between gaze perception and schizotypy [13]. Higher self-referential tendency (lower threshold, for forward faces only) was associated with higher levels of autistic traits (consistent with previous findings [68]) and, interestingly, with higher empathy. This may be because a stronger tendency to feel that social cues are self-directed increases the sensitivity to others’ internal state and thus the ability to empathize. It is worth noting that these relations were only modest in strength. This is not surprising given that these psychological measures tap into higher-order social processes that typically involve multiple components (e.g., autistic traits capture aberrations in attention to detail as well as social difficulties [69]). Nevertheless, the fact that a simple Eye Contact Detection Task could reveal information about functional capacity in more distal, complex socio-emotional domains provides further support that gaze perception is an important social cognitive function. Further research is warranted to replicate these findings and delineate the nature of this relation.

One important question about the construct validity of the eye contact perception measures is whether they measure distinct constructs. We approached this question by examining: 1) if slope and threshold explained unique variance in important social cognitive functions; and 2) if these two metrics provide different/additional/complementary information about eye contact perception when they are derived using forward or deviated faces. The results of the hierarchical regression analyses provided some insight about both—performance on simple (ER-40) and complex (RME) emotion recognition was best explained by different combinations of the two gaze perception metrics (slope and threshold) derived using different head orientations. This supports not only that slope and threshold are tapping into distinct (though moderately correlated) constructs, but also that eye contact perception measures derived from deviated faces provide somewhat different information about one’s social cognition than ones derived using forward faces. Why is this? This may be because head orientation alone is a strong cue of the direction of the gazer’s attention [39,70,71] and significantly influences perceived gaze direction [27,33,38,42,43]. When head orientation is in incongruent direction as gaze direction, uncertainty about gaze direction is increased. As we can see in Table 2, people generally have lower slope (i.e., perceptual precision) when viewing deviated versus forward faces. Our hierarchical analysis finding suggests that higher perceptual precision in the deviated face condition (which suggests more efficient resolution of this uncertainty) predicts better performance on both simple and complex emotion recognition. Taken together, our data suggest that measuring eye contact perception using stimuli depicting deviated head orientations (in addition to forward-facing ones) is better able to capture cognitive processes important to higher-level social cognition.

Finally, the possible influences of sex and age on eye contact perception were explored. Relative to women, men showed reduced perceptual precision for forward faces but increased perceptual precision for deviated faces during eye contact detection. Previous reports found greater variability in men than women during eye contact perception [72,73]. Our results indicate that this may only emerge in the context of forward facing stimuli, suggesting a possible systematic difference in the way men and women respond to head orientation cues in the context of gaze discrimination. Future study is needed to replicate this finding and explore the mechanisms driving this sex difference. In terms of age effects, we found that older individuals exhibited greater perceptual precision than younger participants. This result is the opposite of what would be expected, considering the well-established vision decline associated with aging [74,75]. However, it is possible that deteriorated eyesight among older participants propelled them to sit closer to the screen than younger participants did, thus resulting in better performance in terms of perceptual precision. We should caution that this may be a spurious finding as it did not survive correction for multiple comparisons. Further investigation in more controlled environments is needed to clarify potential age effects in eye contact perception.

To conclude, this study provided evidence for the usefulness of the online psychophysical Eye Contact Detection Task as a tool to measure and understand eye contact perception. Our data provided preliminary support for the reliability and validity of the eye contact perception metrics derived from this task, and informed specific steps to refine the tool and measurement in future investigations. The findings of this study build upon previous work [6] to provide support for future research to further advance our understanding of eye contact perception and uncover its role and mechanisms in social development in children as well as social dysfunction in psychopathology. For example, researchers may use this online tool to conduct a large-scale investigation in clinical populations with known deficits in social cognition such as schizophrenia [9–11], autism [5], and social anxiety [6–8], delineating potential differential abnormalities in perceptual precision and self-referential bias between these disorders. Furthermore, additional study is needed to characterize sex differences in gaze processing and understand the mechanisms. Such knowledge would not only advance our understanding of psychiatric disorders, but also inform the development of targeted interventions to improve social cognition and functioning.

## Supporting information

S1 Fig Relationships between participant age and measures of eye contact perception. Significant relations are indicated using asterisks. None of the associations remained significant after correction for multiple comparisons (4 tests) using Benjamini-Hochberg procedure at FDR of .05. Slope = perceptual precision during eye contact detection; threshold = self-referential tendency during eye contact detection (higher thresholds indicate lower self-referential tendency); *uncorrected *p* < .05.
(TIF)

S2 Fig Scatterplots of associations between measures of eye contact perception, social cognition, and related psychological traits. Slope = perceptual precision during eye contact detection; threshold = self-referential tendency during eye contact detection (higher thresholds indicate lower self-referential tendency); ER-40 = Penn Emotion Recognition Task (accuracy); RME = Reading the Mind in the Eyes Test (accuracy); ASQ = Autism Spectrum Quotient (total score); QCAE = Questionnaire of Cognitive and Affective Empathy (total score); RTS = Referential Thinking Scale (total score). Asterisks indicate uncorrected *p*-values: *** *p* < .001 **p < .01 *p < .05. All correlations with asterisks remained significant at FDR of < .10.
(TIF)

S1 Table Characteristics of the sample at Phase I (*N =* 299) broken down by age group and sex. (DOCX)

S2 Table Descriptive statistics of goodness of fit for final analysis samples. (DOCX)

S3 Table Characteristics of the normative sample: Eye contact perception descriptives by age group and sex. (DOCX)

S4 Table Intra-correlations among eye contact perception measures. (DOCX)

S1 Data (CSV)

S2 Data (CSV)

S3 Data (CSV)

S1 File Image-morphing process for face stimuli. (DOCX)
