# Supplementary figures and images for: Deconstructing eye contact perception: Measuring perceptual precision and self-referential tendency using an online psychophysical eye contact detection task
Source: PLoS One. 2020 Mar 13;15(3):e0230258. doi: 10.1371/journal.pone.0230258 (PMC7069644; doi:10.1371/journal.pone.0230258)

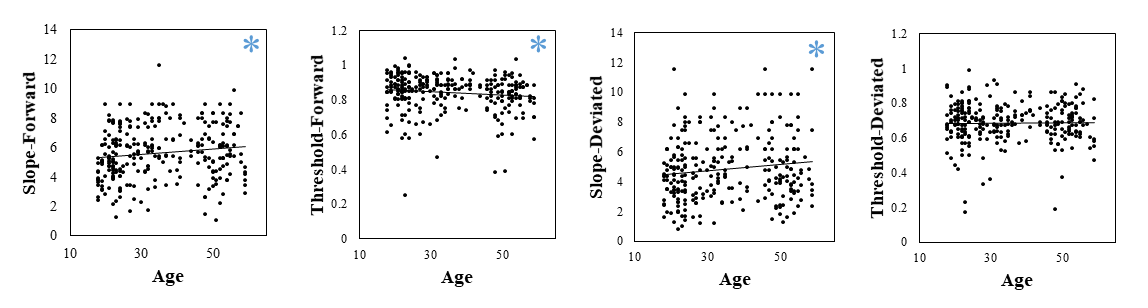

Supplement: S1 Fig — Significant relations are indicated using asterisks. None of the associations remained significant after correction for multiple comparisons (4 tests) using Benjamini-Hochberg procedure at FDR of .05. Slope = perceptual precision during eye contact detection; threshold = self-referential tendency during eye contact detection (higher thresholds indicate lower self-referential tendency); *uncorrected p < .05. (TIF) [file pone.0230258.s001.tif]

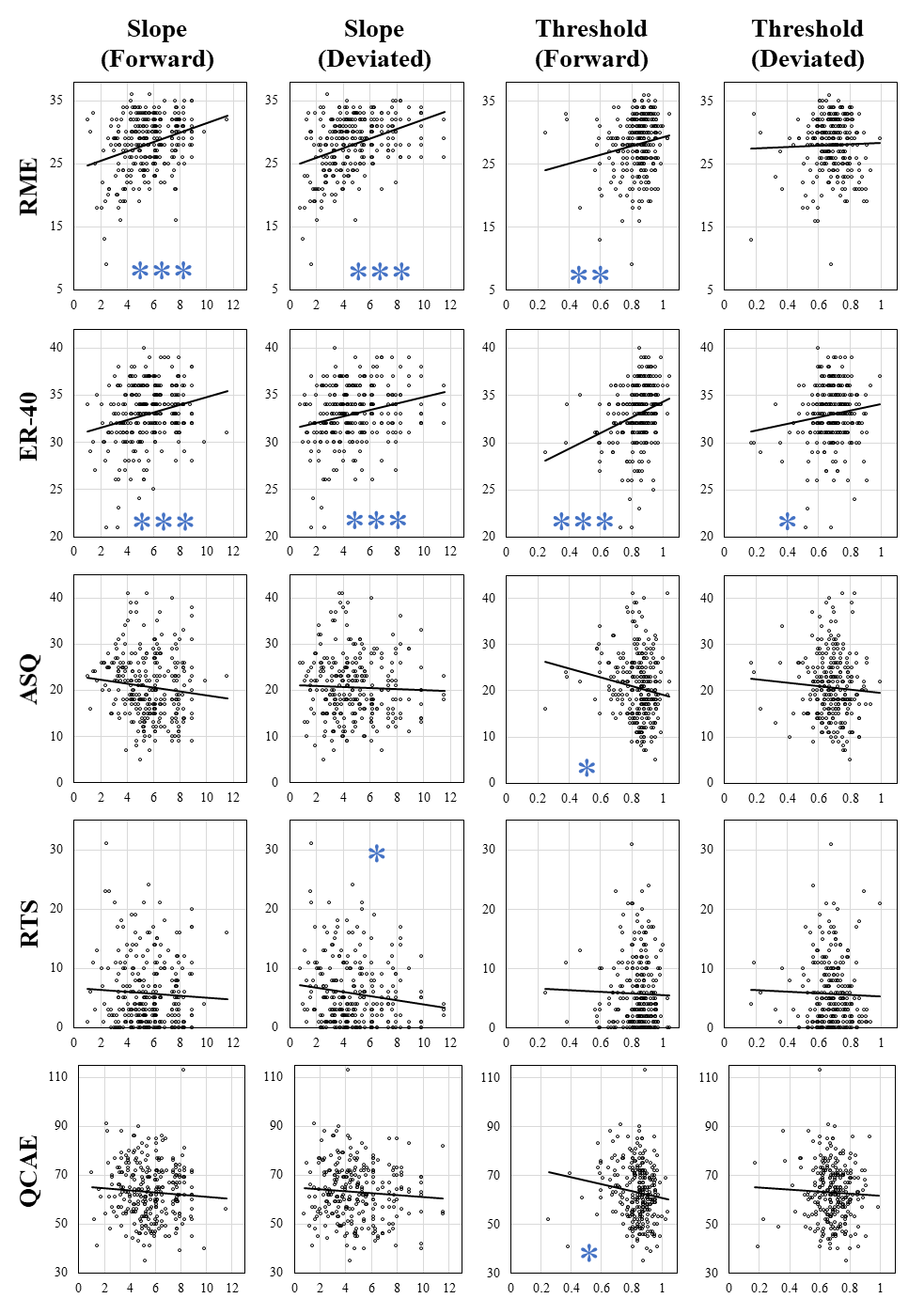

Supplement: S2 Fig — Slope = perceptual precision during eye contact detection; threshold = self-referential tendency during eye contact detection (higher thresholds indicate lower self-referential tendency); ER-40 = Penn Emotion Recognition Task (accuracy); RME = Reading the Mind in the Eyes Test (accuracy); ASQ = Autism Spectrum Quotient (total score); QCAE = Questionnaire of Cognitive and Affective Empathy (total score); RTS = Referential Thinking Scale (total score). Asterisks indicate uncorrected p-values: *** p < .001 **p < .01 *p < .05. All correlations with asterisks remained significant at FDR of < .10. (TIF) [file pone.0230258.s002.tif]
