# Supplementary material for: Deconstructing eye contact perception: Measuring perceptual precision and self-referential tendency using an online psychophysical eye contact detection task
Source: PLoS One. 2020 Mar 13;15(3):e0230258. doi: 10.1371/journal.pone.0230258 (PMC7069644; doi:10.1371/journal.pone.0230258)
Supplement: S1 Table — (DOCX) [file pone.0230258.s003.docx]

**Table S1. Characteristics of the sample at Phase I (*N =* 299) broken down by age group and sex.**

| **Age Group** | **18-25** | | **26-45** | | **46-60** | | **All** | | |
| --- | --- | --- | --- | --- | --- | --- | --- | --- | --- |
|  | **Male** **(*n* = 52)** | **Female** **(*n* = 50)** | **Male** **(*n* = 49)** | **Female** **(*n* = 48)** | **Male** **(*n* = 50)** | **Female** **(*n* = 50)** | **Male** **(*n* = 151)** | **Female** **(*n* =148)** |  |
|  | **M (SD)** | **M (SD)** | **M (SD)** | **M (SD)** | **M (SD)** | **M (SD)** | **M (SD)** | **M (SD)** |  |
| **Age** | 21.2 (2.13) | 21.8 (1.86) | 32.0 (4.46) | 34.0 (5.16) | 51.7 (3.66) | 51.9 (3.88) | 34.8 (13.21) | 35.92 (13.04) |  |
| **Education** | 14.0 (2.05) | 14.7 (2.18) | 15.0 (2.24) | 15.2 (2.33) | 15.3 (2.53) | 14.9 (2.53) | 14.74 (2.34) | 14.95 (2.34) |  |
| **Parental Education** | 14.6 (2.96) | 14.3 (2.63) | 14.3 (2.81) | 13.8 (2.29) | 14.0 (2.44) | 12.9 (2.37) ^a^ | 14.29 (2.74) | 13.69 (2.49) ^a^ |  |
| **Race** | ***n* (%)** | ***n* (%)** | ***n* (%)** | ***n* (%)** | ***n* (%)** | ***n* (%)** | ***n* (%)** | ***n* (%)** |  |
| White | 40 (76.9) | 39 (78.0) | 42 (85.7) | 40 (83.3) | 47 (94.0) | 46 (92.0) | 129 (85.4) | 125 (84.5) |  |
| Black | 2 (3.8) | 3 (6.0) | 3 (6.1) | 3 (6.3) | 1 (2.0) | 1 (2.0) | 6 (4.0) | 7 (4.7) |  |
| Asian | 9 (17.3) | 8 (16.0) | 4 (8.1) | 4 (8.4) | 2 (4.0) | 1 (2.0) | 15 (9.9) | 13 (8.8) |  |
| Other/NR | 1 (1.9) | 0 (0) | 0 (0) | 1 (2.1) | 0 (0) | 2 (4.0) | 1 (0.7) | 3 (2.0) |  |

*Note.* NR = Prefer not to respond; Education = years of education completed; Parental education = highest education completed by either parent (in years); ^a^ Data on parental education missing for one female participant in 46-60 age group.
