# Supplementary material for: Deconstructing eye contact perception: Measuring perceptual precision and self-referential tendency using an online psychophysical eye contact detection task
Source: PLoS One. 2020 Mar 13;15(3):e0230258. doi: 10.1371/journal.pone.0230258 (PMC7069644; doi:10.1371/journal.pone.0230258)
Supplement: S2 Table — (DOCX) [file pone.0230258.s004.docx]

**Table S2. Descriptive statistics of goodness of fit for final analysis samples.**

|  | **Forward Faces** | | **Deviated Faces** | |
| --- | --- | --- | --- | --- |
|  | ***n*** | **M (SD)** | ***n*** | **M (SD)** |
| *Full analysis sample* |  |  |  |  |
| Phase I: Deviance residuals | 282 | 2.6 (3.0) | 269 | 5.2 (4.4) |
| *Test-retest reliability sample* |  |  |  |  |
| Time 1: Deviance residuals | 115 | 2.4 (2.6) | 106 | 4.7 (4.4) |
| Time 2: Deviance residuals | 112 | 2.8 (2.5) | 104 | 4.5 (4.2) |

*Note.* Values indicate means (standard deviations) of deviance residuals from the fitted logit functions to eye contact perception data. Higher deviance residuals indicate greater discrepancy between model fit and data.
