# Supplementary material for: Deconstructing eye contact perception: Measuring perceptual precision and self-referential tendency using an online psychophysical eye contact detection task
Source: PLoS One. 2020 Mar 13;15(3):e0230258. doi: 10.1371/journal.pone.0230258 (PMC7069644; doi:10.1371/journal.pone.0230258)
Supplement: S3 Table — (DOCX) [file pone.0230258.s005.docx]

**Table S3. Characteristics of the normative sample: Eye contact perception descriptives by age group and sex.**

| **Age Group** | **18-25** | | | | | **26-45** | | | | **46-60** | | | | | **All** | | | | |
| --- | --- | --- | --- | --- | --- | --- | --- | --- | --- | --- | --- | --- | --- | --- | --- | --- | --- | --- | --- |
|  | **Male** | | **Female** | | **Male** | | | **Female** | | **Male** | | | **Female** | | | **Male** | | **Female** | |
|  | ***n*** | ***M (SD)*** | ***n*** | ***M (SD)*** | ***n*** | | ***M (SD)*** | ***n*** | ***M (SD)*** | ***n*** | ***M (SD)*** | ***n*** | | ***M (SD)*** | ***n*** | | ***M (SD)*** | ***n*** | ***M (SD)*** |
| *Eye Contact Perception Measures* | | | | | | | | | | | | | | | | | | | |
| Slope-Forward | 48 | 4.8 (1.6) | 47 | 5.5 (1.6) | 47 | | 5.6 (1.8) | 46 | 6.4 (1.8) | 46 | 5.6 (1.7) | 48 | | 5.9 (2.0) | 141 | | 5.33 (1.7) | 141 | 5.9 (1.8) |
| Slope-Deviated | 46 | 4.2 (2.2) | 46 | 4.7 (2.0) | 43 | | 4.9 (2.1) | 43 | 5.2 (2.1) | 46 | 5.6 (2.6) | 45 | | 4.4 (1.9) | 135 | | 4.91 (2.3) | 134 | 4,8 (2.0) |
| Threshold-Forward | 48 | 0.8 (0,1) | 47 | 0.9 (0.1) | 47 | | 0.9 (0.1) | 46 | 0.9 (0.1) | 46 | 0.8 (0.1) | 48 | | 0.8 (0.1) | 141 | | 0.8 (0.1) | 141 | 0.9 (0.1) |
| Threshold-Deviated | 46 | 0.7 (0.2) | 46 | 0.7 (0.1) | 43 | | 0.7 (0.1) | 43 | 0.7 (0.1) | 46 | 0.7 (0,1) | 45 | | 0.7 (0.1) | 135 | | 0.7 (0.1) | 134 | 0.7 (0.1) |

*Note.* Based on data from the full sample at Phase I (*N =* 299). Slope = perceptual precision during eye contact detection for forward or deviated faces (higher slope values indicate greater perceptual precision); threshold = self-referential tendency during eye contact detection for forward or deviated faces (higher thresholds indicate lower self-referential tendency).
