# Supplementary material for: Deconstructing eye contact perception: Measuring perceptual precision and self-referential tendency using an online psychophysical eye contact detection task
Source: PLoS One. 2020 Mar 13;15(3):e0230258. doi: 10.1371/journal.pone.0230258 (PMC7069644; doi:10.1371/journal.pone.0230258)
Supplement: S4 Table — (DOCX) [file pone.0230258.s006.docx]

**Table S4. Intra-correlations among eye contact perception measures.**

|  | **Slope** | | | | **Threshold** | | | |
| --- | --- | --- | --- | --- | --- | --- | --- | --- |
|  | Forward Faces | | Deviated Faces | | Forward Faces | | Deviated Faces | |
|  | ***n*** | ***r*** | ***n*** | ***r*** | ***n*** | ***r*** | ***n*** | ***r*** |
| Slope-Forward | 282 |  |  |  |  |  |  |  |
| Slope-Deviated | 256 | .456*** | 269 |  |  |  |  |  |
| Threshold-Forward | 282 | .457*** | 256 | .308*** | 282 |  |  |  |
| Threshold-Deviated | 256 | .229*** | 269 | .075 | 256 | .599*** | 269 |  |

*Note.* Based on data from the sample at Phase I. Slope = perceptual precision during eye contact detection; threshold = self-referential tendency during eye contact detection (higher thresholds indicate lower self-referential tendency). ***uncorrected *p* < .001.
