# Supplementary material for: Deconstructing eye contact perception: Measuring perceptual precision and self-referential tendency using an online psychophysical eye contact detection task
Source: PLoS One. 2020 Mar 13;15(3):e0230258. doi: 10.1371/journal.pone.0230258 (PMC7069644; doi:10.1371/journal.pone.0230258)
Supplement: S1 File — (DOCX) [file pone.0230258.s010.docx]

**Supporting Information S1. Image-morphing process for face stimuli.**

Stimuli were created by morphing a set of original images from George, Driver and Dolan (44) using Abrosoft Fanta Morph software (Beijing, China). Initially, original image sets for each actor consisted of only four images (see Fig 1): (1) direct eye contact, head forward, (2) direct eye contact, head “deviated” (i.e., turned 30° away from viewer), (3) eyes “averted” (i.e., looking 30° left of the viewer), head forward, and (4) eyes averted, head deviated. Findings from our previous study—using the same task structure, but a different set of images—suggested that 11 stimulus strengths represented by gaze angles from direct to 30° averted was a satisfactory number of sampling points which would center the logistic curve near a stimuli strength of 0.5 (11). First, forward-facing images (direct eye contact, eyes 30° to the left) for each actor were morphed together using Fanta Morph to create 9 new images exhibiting more-precise, incremental gaze angles between the original 2 images. This resulted in a set of 11 images for each actor displaying gaze from direct eye contact (0°) to left-most averted (eyes averted left 30°) in 10% increments (0°, eyes looking 3° left, 6° left, and so on; see Fig 1). Next, these image sets were duplicated and flipped horizontally such that the resulting images exhibited right-averted gaze in the same increments. Lastly, the same process was completed again using deviated head images for all actors. The result was a final set of 264 stimuli, which consisted of images exhibiting: 6 actors (3 male, 3 female) × 2 head orientations (forward, deviated) × 11 eye contact signal strengths (eyes averted 0°, 3°, 6°, 9°, 12°, 15°, 18°, 21°, 24°, 27°, and 30°) × 2 gaze directions (eyes averted leftward, eyes averted rightward). Examples of the original images and the resulting morphed images for one actor are provided in Figure 1.
